# Glioblastoma infiltration of both tumor- and virus-antigen specific cytotoxic T cells correlates with experimental virotherapy responses

**DOI:** 10.1038/s41598-020-61736-2

**Published:** 2020-03-20

**Authors:** Quazim A. Alayo, Hirotaka Ito, Carmela Passaro, Mykola Zdioruk, Ahmad Bakur Mahmoud, Korneel Grauwet, Xiaoli Zhang, Sean E. Lawler, David A. Reardon, William F. Goins, Soledad Fernandez, E. Antonio Chiocca, Hiroshi Nakashima

**Affiliations:** 1 Harvey W. Cushing Neuro-oncology Laboratories (HCNL), Department of Neurosurgery, Harvard Medical School and Brigham and Women’s Hospital, 02115 Boston, MA USA; 2 Center for Biostatistics, Department of Biomedical Informatics, The Ohio State University, 43210 Columbus, OH USA; 3 Center for Neuro-Oncology, Dana-Farber Cancer Institute, 02115 Boston, MA USA; 4 Department of Microbiology and Molecular Genetics, University of Pittsburgh School of Medicine, 15219 Pittsburgh, PA USA; 5 Present Address: College of Applied Medical Sciences, Taibah University, 42353 Madinah, Saudi Arabia; 6 Present Address: Cancer Center and Department of Medicine, Massachusetts General Hospital, Boston, 02114 MA USA; 7 Present Address: Department of Internal Medicine, St. Luke’s hospital, 63017 Chesterfield, MO USA

**Keywords:** Tumour immunology, Preclinical research, Translational research

## Abstract

The mode of action for oncolytic viruses (OVs) in cancer treatment is thought to depend on a direct initial cytotoxic effect against infected tumor cells and subsequent activation of immune cell responses directed against the neoplasm. To study both of these effects in a mouse model of glioblastoma (GBM), we employed murine GBM cells engineered to constitutively express the type I Herpes Simplex Virus (HSV1) HSV-1 receptor, nectin-1, to allow for more efficient infection and replication by oncolytic HSV (oHSV). These cells were further engineered with a surrogate tumor antigen to facilitate assays of T cell activity. We utilized MRI-based volumetrics to measure GBM responses after injection with the oHSV and bioluminescent imaging (BLI) to determine oHSV replicative kinetics in the injected tumor mass. We found increased infiltration of both surrogate tumor antigen- and oHSV antigen-specific CD8+ T cells within 7 days after oHSV injection. There was no increase in tumor infiltrating CD8+ T cells expressing “exhaustion” markers, yet oHSV infection led to a reduction in PD-1+ CD8+ T cells in injected GBMs and an increase in IFN*γ*+ CD8+ T cells. There was a significant direct correlation between oHSV-mediated reduction in GBM volume and increased infiltration of both viral and tumor antigen-specific CD8+ T cells, as well as oHSV intratumoral gene activity. These findings imply that CD8+ T cell cytotoxicity against both tumor and viral antigens as well as intratumoral oHSV gene expression are important in oHSV-mediated GBM therapy.

## Introduction

There is resurgent interest in the utilization of oncolytic viruses (OVs) for cancer therapy, based on results of several advanced clinical trials including one that has led to FDA approval of an oncolytic herpes simplex virus (oHSV) for the treatment of advanced melanoma^1–3^. The mode of action of OVs is thought to be based on an initial direct cytotoxic effect by the infecting virus leading to immunogenic cell death, a second phase of viral replication and propagation that bio-distributes virus-induced cytotoxicity to multiple cells within the neoplastic mass, and a third phase of activation of innate and adaptive immune responses against tumor antigens but also against viral antigens^4^. The induction of an adaptive immune response against tumor cells is thought to be the most important mode of action for OVs to sustain durable antitumor efficacy. In addition to an anti-viral response, to be effective, this immune response should be directed against tumor antigens, requiring functional cytotoxic CD8+ T cell recognition of neoplastic epitopes. So far, the interplay between CD8+ T cell recognition of tumor antigens vs. viral antigens and how this affects antitumor efficacy has not been explored extensively. Moreover, the relative contribution of the viral oncolytic effect versus the immune response against tumor and/or virus in virotherapy efficacy is still debated^5^.

Oncolytic viruses based on HSV-1 are amongst the most widely studied and have been tested in several clinical trials^2,5–15^. In addition, several preclinical studies have been performed in mouse models of cancer. Unfortunately, dissection of the relative contribution and importance of the different modes of anticancer action of oHSV is limited by oHSV tropism for human vs. mouse tumor cells. Several studies have been focused on understanding how to increase oHSV infection, replication and propagation in human tumors established in immunodeficient mouse models but these fail to assess the effect of CD8+ T cells, and other adaptive immune components against tumors^4^. Conversely, in immunocompetent mouse tumor models, oHSV infection and replication in many mouse tumors becomes severely limited and thus these studies focus on ways to increase oHSV-mediated immunogenicity of tumors without being able to fully understand the implications of viral infection, replication, propagation and oHSV biodistribution within the tumor mass in immune competent mice^8^.

In mouse models of cancer, the traditional outcome measure of therapy effectiveness is based on increase in overall long-term animal survival. However, in humans with cancer, therapy effectiveness is also measured by changes in volumetric radiologic magnetic resonance imaging (MRI). Often, this imaging modality provides a short-term measure of therapy success. Yet, this outcome measure is not routinely utilized in preclinical models of cancer therapeutics.

Based on the above considerations, we hypothesized that the success of oHSV-mediated therapy against cancer in immune competent mice correlates with an increase of intratumoral antigen-specific and functional infiltrating CD8+ cytotoxic T cells. To test this, we engineered mouse GBM cells that can be better infected by oHSV and that also express a surrogate neoantigen^16^. We then utilized these GBM cells to show that oHSV intra-tumoral administration leads to MRI evidence of tumor shrinkage (Response) in some mice, but also tumor growth in others (Progression). After oHSV treatment, we could significantly correlate tumor volume reduction with a significant increase in the percentage of tumor antigen-specific, functional CD8+ T cells that infiltrate into the tumor. Somewhat unexpectedly because of possible occurrence of immunodominance by viral epitopes^17^, tumor infiltration of viral antigen-specific CD8+ T cells also significantly correlated with changes in tumor volumes after oHSV therapy. There was also a significant correlation in other immune cell changes and tumor response after oHSV administration. Increased oHSV-based gene expression (suggestive of more replicative biodistribution) also correlated with smaller tumor volumes. Together, these results provide evidence that tumor responsiveness to oHSV as measured by reduction in MRI tumor volumes correlates with complex changes in the immune cell microenvironment, as well as increased viral gene expression.

## Results

### Engineering mouse GBM cells that can be readily infected by oHSV and that express a surrogate tumor antigen

We initially screened several available mouse GBM cells, without finding a suitable cell that allowed for high-level oHSV replication. We had previously shown that expression of the major human HSV1 receptor (Nectin-1/PVRL1/HVEC/CD111)^18^ in mouse GL261 GBM cells increased the capacity of oHSV to infect and replicate within this mouse GBM line^19^. Based on this result, we engineered mouse CT2A GBM cells to express Nectin-1 (Fig. S1a). These cells are infected by oHSV and allow for higher virus replication than parental cells and approach levels measured in human GBM cells (Figs. S1b,c and 1). Although these murine GBM cells express a human HSV1 receptor, they still establish in tumors in the brains of syngeneic C57Bl/6 mice (Fig. S1d)^19,20^. These data thus suggest that GL261nectin1 or CT2Anectin1 can be used to study both the direct cytotoxic effect of the oHSV and the subsequent inflammatory and cytotoxic T cell response against tumor antigens presented in the context of an active oHSV infection in C57Bl/6 mouse glioma models.

**Figure 1:**
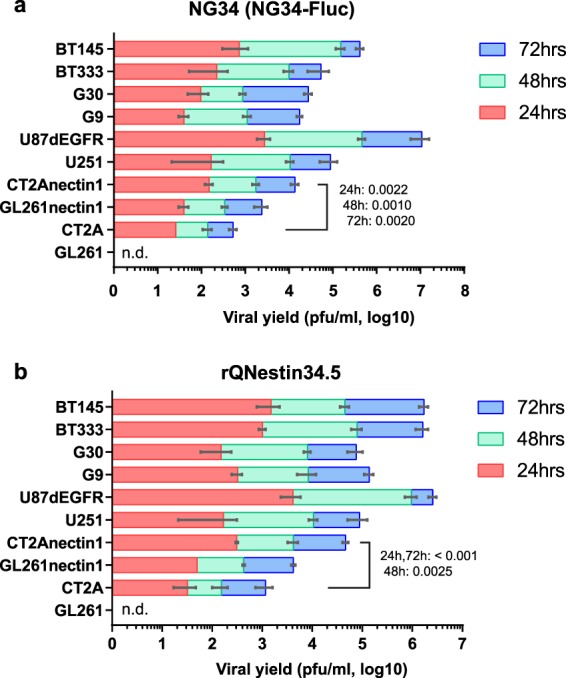
Replication kinetics of two oncolytic HSVs (**a**; NG34-Fluc, **b**; rQNestin34.5) in mouse and human GBM cells. The day after plating cells, oHSV were added at a MOI of 0.1. Stacked bars show viral yields at 24 (red), 48 (green), and 72 hours (blue). GL261 and CT2A are parental mouse GBM cells, while GL261nectin1 and CT2Anectin1 express the HSV-1 receptor Nectin-1. Human U251, U87*Δ*EGFR are established human GBM lines which are readily infected by oHSV, while G9, G30, BT333, and BT145 are patient-derived human GBMs grown under GBM stem-like conditions. Error bars shows SD (n = 3).Values on the stacked bars show P-values with Holm-Sidak t-test of CT2A vs CT2Anectin1 at 24, 48 and 72 hours. n.d. = not detected.

We next sought to characterize changes in tumor and oHSV specific CD8+ T-cell responses following oHSV injection into tumors in brain. Since neither GL261 nor CT2A tumor antigens have been well characterized, we needed to utilize GBM cells, engineered to express the GP33 (gp33-41), a model tumor antigen from Lymphocytic Choriomeningitis Virus (LCMV) glycoprotein to measure cytotoxic CD8+ T cells specific to tumor (GP33) and HSV antigens (gB498-505) in both blood and in the brain of mice using peptide-MHC-I (pMHC) tetramers^16,21^. In addition to these modifications to tumor cells to allow for cytotoxic T cell assays, we also engineered the oHSV to express firefly luciferase (Fluc) to measure viral replicative kinetics and engineered tumor cells to express Renilla luciferase (Rluc) to assay tumor viability with optical bioluminescent imaging (BLI)^22^.

### Magnetic resonance and bioluminescence imaging to assay for mouse GBM response to oHSV therapy

To correlate oHSV treatment with tumor response or lack thereof, we employed both MRI and BLI. MRI is routinely used to measure GBM responses clinically. We then assessed mice whose tumor volume decreased (“responder”-stable or reduced tumor volume) and those whose tumor continued to grow (“non-responder”- increased tumor volume) (Fig. S2). For each mouse, we assayed tumor volume by MRI, oHSV-mediated gene expression by Fluc, and tumor viability by Rluc, as shown in the examples provided in Fig. S2. A total of three independent experiments were performed altering variables such as number of injected tumor cells, injected oHSV doses or control and/or time to assay for effects, as summarized in Fig. S3a, as explained in more detail below.

**Figure 2:**
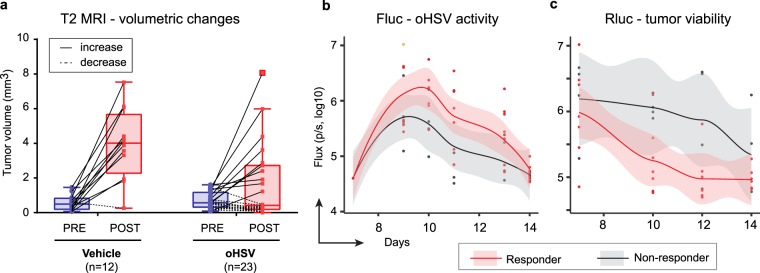
Analysis of oHSV treatment responders vs. non-responders. (**a**) MRI-measured tumor volumes are plotted with bars separately for vehicle (PBS) and oHSV treatment groups. Tumor volumes before (PRE; blue) and after treatment (POST; red) are plotted with a solid line for increased volumes or dotted line for decreased or unchanged volumes. Individual experimental data and MRI schedule are detailed in Fig. S3,b, e,h. Optical bioluminescent images were captured before and after injection of the appropriate substrate for firefly luciferase (Fluc; **b**) and renilla (Rluc; **c**) at multiple-time points in experiment 2. Scatter plots (dot), non-linear regression curve (solid line) and two-sided 95% confidential intervals (shadow) are shown for each graph. oHSV treatment mice were divided in two groups based on tumor responses by MRI from panel a: Non-responder (gray shadow with solid black line) and responder (light red shadow with solid red line). BLI and MRI results of Individual mouse on the time-course are also shown in Fig. S3.

The combined MRI-measured tumor volumetrics of these three experiments is summarized in Fig. 2a. Ninety-two percent (92%, 11 out of 12) of tumors grew after intratumoral vehicle injection, while 43% (10/23) of tumors grew after oHSV treatment. The BLI (Fluc and Rluc) assays were more complex: unexpectedly, when taken altogether, there appeared to be no correlation between responders and non-responders in the temporal kinetics of Fluc and Rluc over time (data not shown). However, when the three experiments were analyzed separately, experiment 2 showed a significant divergence in the Fluc (oHSV) (Fig. 2b) vs. Rluc (tumor) (Fig. 2c) signal in the responder vs. non-responder mice but this was not observed in experiment 3 and experiment 1 (data not shown). It should be noted that there were no responders in experiment 1 and this was expected.

As detailed above, Fig. S3a lists the details for each experiment. In the first experiment, 3 × 10^5^ CT2Agp33nectin1 tumor cells were implanted in mouse brains (day 0). On day 6, an MRI was performed for initial volumetric analysis and then on day 7, oHSV (1 × 10^6^ pfu) was administered. An MRI to assess response was performed on day 13 (6 days post oHSV injection), before euthanasia on day 14 (Fig. S3b). The results show that 4/4 (100%) control tumors and all 7 (100%) oHSV treated tumors (Fig. S3b) grew, although there was a very small increase in tumor volumes (under 2mm3) in 2/7 of oHSV-treated mice. BLI was performed the day before oHSV injection and on multiple days after. Figure S3c,d show the temporal kinetics for each imaged mouse: there was an initial increase in Fluc (oHSV) activity, which then gradually decreased over time (Fig. S3c), but Rluc activity (tumor) tended to stay the same or grow in oHSV-treated mice (Fig. S3d).

Since there were almost no responders from this experiment, we modified the initial cell inoculum and number of injected oHSV. In the second experiment, 2 × 10^5^ CT2Agp33nectin1 tumor cells were implanted in mouse brains (day 0). After an MRI on day 7 for baseline tumor volumes, 2 × 10^6^ pfu of oHSV were stereotactically injected intratumorally. An MRI was obtained at day 14 to measure tumor volume, followed by brain harvesting on day 15. We repeated the same experiment to ascertain temporal kinetics of immune cell infiltrates on day 11. Figure S3e shows that tumor growth occurred in 5/5 (100%) mice injected with vehicle, while only 4/11 (36%) tumors treated with oHSV grew (i.e., non-responders). The Fluc (oHSV) BLI showed an initial increase in oHSV mediated gene activity with a gradual decrease over time (Fig. S3f). The Rluc (tumor) BLI showed a temporal decrease in activity in the oHSV-treated vs. control group (Fig. S3g).

In the third experiment, we asked if the presence or absence of a tumor response after virotherapy was detected even at later time points. Similar to experiment 2, 2 × 10^5^ CT2Agp33nectin1 tumor cells were implanted in mouse brains on day 0. After an MRI on day 6, oHSV (2 × 10^6^ pfu) was injected on day 7. We delayed post-treatment MRI acquisition until day 16 and mice were sacrificed for immune cell analyses at day 20. Figure S3h shows 2/3 control tumors grew (66.6%), while only 2/5 (40%) oHSV treated tumors grew. The Fluc (oHSV) BLI showed an initial increase in oHSV mediated gene activity with a gradual decrease over time (Fig. S3i). The Rluc (tumor) BLI showed no differences in activity in the oHSV-treated vs. control group (Fig. S3j). Of note, pre-treatment tumor volumes as assessed by MRI are similar in experiment 1 and experiments 2 and 3 (Fig. S4).

Taken together, these results showed variability in tumor responses to oHSV, although a reduced tumor cell inoculum, increased number of oHSV pfu injected, and a 7-day time frame for evaluation (experiment 2 vs. 1 and 3) increased the number of responders. The estimation of the number of mice responding to the tumor cell injections were more consistent using MRI compared to BLI as an imaging modality.

### Cytotoxic T cell assays against oHSV vs. tumor antigen

**Figure 3:**
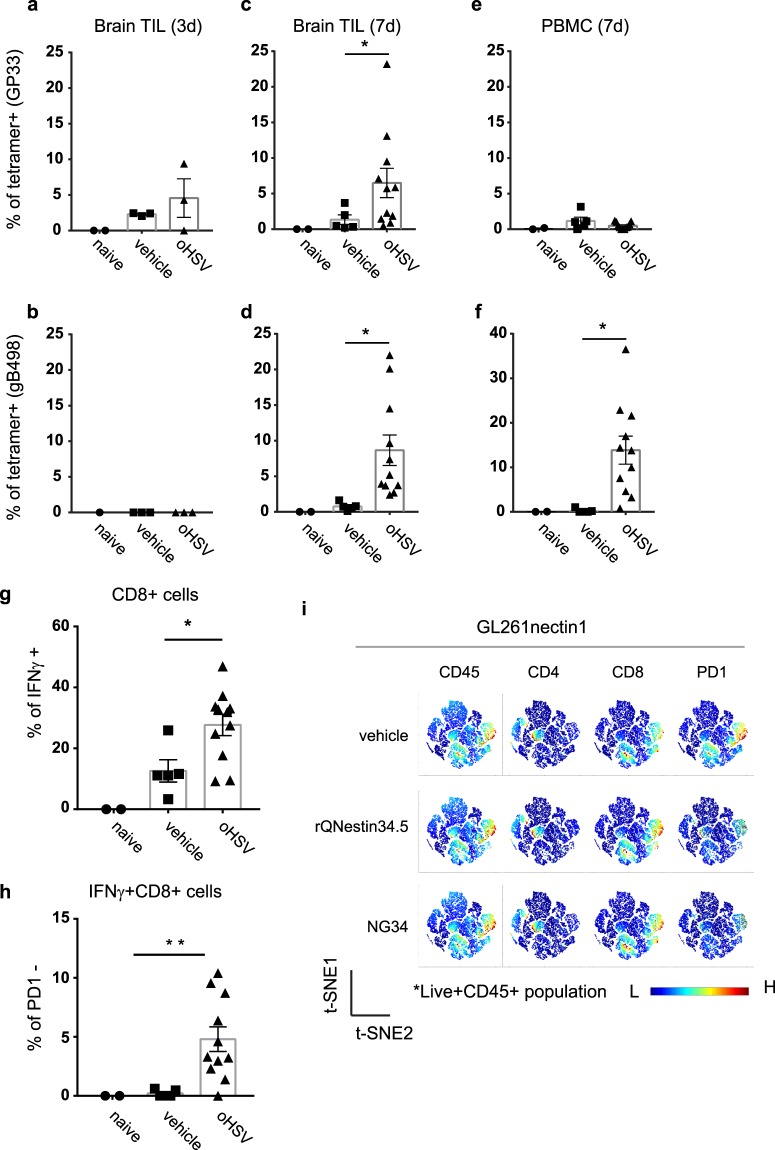
Immune cell analyses. (**a**–**f**) CD8+ T cells against GP33 (GBM antigen; panel a,c,e) or gB498 (oHSV antigen; panel b,d,f), 3 (left panels) or 7 (middle and right) days after oHSV or PBS injection in GBMs (labeled as CT2Agp33nectin1) implanted in mouse brains. CD8+ T cells were gated from CD45+TCR*β*+ population isolated by Percoll gradient-enriched leukocytes from excised brains (Brain TIL) and PBMCs. GP33 or gB498+ CD8+ T-cells were stained with corresponding tetramers. Controls were from tumor-free native mouse brains (Naïve) compared to vehicle or oHSV injected mouse brains. Each dot represents an individual mouse sample. The bars represent the mean and the range represents the SEM. (**g**) Percent of GBM-infiltrating whole CD8+ T cells and (**h**) PD-1-negative CD8+ T cells that express IFN*γ*, 7 days after oHSV injection. *p < 0.05, one-way ANOVA; (**i**) Unbiased immune marker analysis by t-Distributed Stochastic Neighboring viSNE revealed PD-1 downregulation on the cytotoxic CD8+ T cells in brains from murine GL261nectin1 GBM-bearing C57Bl/6 mice after oHSV treatment (rQNestin34.5 and NG3420) at day 7 compared to naïve control group.

Next, we asked if oHSV-mediated anti-GBM responses were associated with temporal changes in CD8+ T cells infiltrating the tumor in response to tumor and viral antigens. In the previously described experiment 2, we harvested GBM tumors at 3 and 7 days after oHSV injection and isolated TILs (and PBMCs) to measure different immune cell sub-populations by FACS (T cells in Fig. S5a and myeloid cell in Fig. S5b). oHSV treatment did not produce a significant change in the percentage (Fig. S6a) or total number (Fig. S6b) of CD8+ T cells recruited to the tumor mass. However, there was a significant increase in tumor-infiltrating GP33-tetramer positive tumor antigen-specific CD8+ T cells at 3 (left panels) and 7 (middle) days after oHSV injection when compared to control tumors (Fig. 3a–f). At 3 days, there was an average of 4% GP33-specific CD8+ T cells vs. 2% in controls in brain TILs(Fig. 3a). At 7 days, an average of 7% of GP33-specific CD8+ T cells were detected compared to less than 2% in controls (p = 0.02) in brain TILs. (Figure 3c). This result suggests that *in situ* oHSV treatment promotes tumor-infiltration or proliferation of tumor specific CD8+ T cells. As expected, there was also an increase in CD8+ T cells specific for the oHSV antigen, gB498^23^ (Fig. 3b,d). Specifically, at 7 days this percentage was similar in magnitude to that of GP33+ T cells. There was no GP33+ CD8+ T-cell enrichment in PBMCs, but there was an expansion of gB498+ CD8+ T cells as expected (Fig. 3e,f). There was no increase in T-cell exhaustion markers (PD-1, Tim-3, LAG-3 and TIGIT) in the pan-CD8+ TIL population on day 7 between oHSV-treated and vehicle groups (Fig. S7). These results thus showed that oHSV injection in tumors led to a significant increase in infiltration of cytotoxic CD8+ T cells specific for the GP33 surrogate antigen expressed by GBM cells. There was also an expected increase in infiltration of oHSV-specific cytotoxic T cells.

The tumor-specific CD8+ TIL population was also assessed for IFN*γ* expression, showing significant expansion in the oHSV treated group compared to vehicle controls (Fig. 3g). The oHSV-treated group also exhibited a significant decrease in the PD-1+ sub-population of these IFN*γ* producing CD8+ TILs (Fig. 3h). This observation was also consistent with the GL261nectin1 GBM model where treatment with two different oHSVs (rQNestin34.5 or NG34) decreased PD-1 levels in CD8+ co-localized clusters in an unbiased analysis (Fig. 3i). These data thus suggested that oHSV injection does indeed expand the tumor infiltrating CD8+ T cell population specific for tumor native antigens (Fig. 3g) as well as the surrogate GP33 antigen (Fig. 3c).

#### Significant correlation between MRI-measured tumor volumes after oHSV and GP33-specific and gB-498 CD8+ T cell GBM infiltration

**Figure 4:**
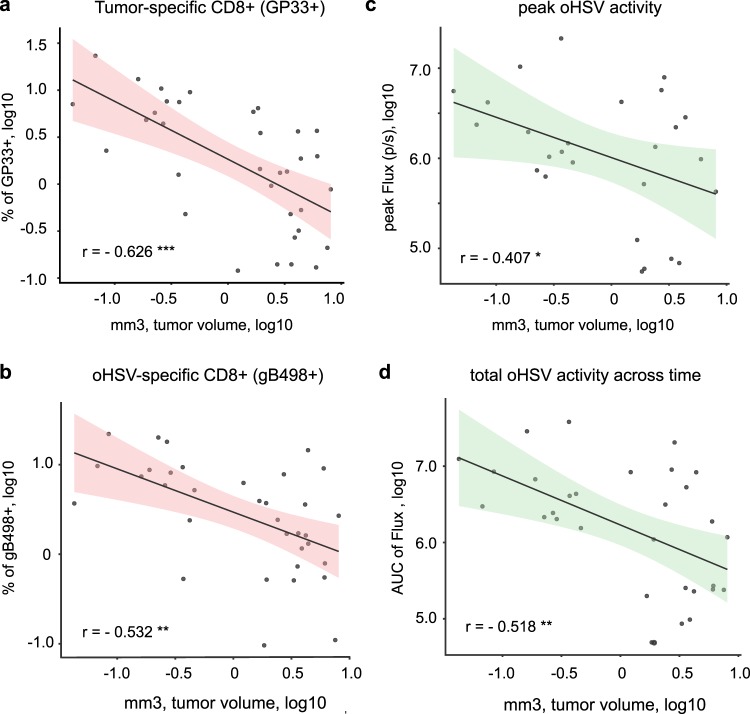
Tumor volume correlations with tumor- and oHSV-specific CD8+ T-cell infiltrates and oHSV gene expression. MRI and BLI data from three separate experiments were combined to generate scatter dot plots and a linear regression line with the two-sided 95% confidence interval (pink or green shadows). Timing of MRI scans and BLI at various times post-treatment in each experiment are summarized in Fig. S3a. Tumor volumes measured by MRI were tested as follows; FACS analyzed data of (**a**) GP33 tetramer+ CD8+TCR*β*+CD45+live+ cell population, (**b**) gB498 tetramer+ CD8+TCR*β*+CD45+live+ cells population, or BLI-based data of (**c**) peak signals of Fluc flux or (**d**) AUC of Fluc across time-points (Fig. S3a). Each plot includes the Pearson’s correlation coefficient (r) and p-value (* < 0.05, **<0.01, *** < 0.001) which are detailed further in Fig. 5.

We then tested whether MRI tumor volumes after oHSV therapy correlated with percentages of surrogate tumor antigen (GP33)- or viral antigen-specific CD8+ TILs. Figure 4a shows that in all 3 experiments there was a significant inverse correlation between the MRI volumes post-treatment (either oHSV or vehicle) and the percentage of GP33+ CD8+ T cells infiltrating mouse GBMs. Surprisingly, there was also a significant correlation between MRI volumes after oHSV treatment, and the percentage of gB498+ CD8+ T cells infiltrating tumors (Fig. 4b). Not surprisingly there was also a significant correlation in the peak FLuc (oHSV activity; Fig. 4c) and total FLuc expression (total oHSV activity across time; Fig. 4d). The sum of these experiments thus validates the hypothesis that MRI-measured volumes correlate with increases in tumor infiltration of tumor antigen-specific CD8+ T cells, as well as increases in viral antigen-specific CD8+ T cells. It also shows that oHSV activity (measured by Fluc) also correlates with volumes.

#### Investigation of additional correlates of tumor response

**Figure 5:**
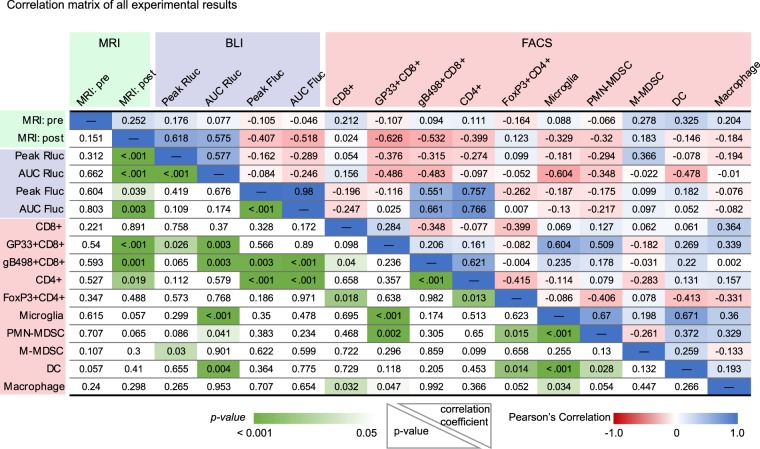
Statistical tests of significance and Pearson’s correlation coefficients between measured variables in the tumor microenvironment. Three independent experiments from MRI, BLI and FACS were aggregated to analyze correlations between two variables. Pearson’s correlation coefficient and p values for each variable against the other is shown in the upper right and lower left of the table, respectively. The color gradient shows the strength of correlation, as direct (blue), inverse (red), and its significance (green). Details are in the Materials and Methods section.

We asked if changes in other immune cells within the TME or viral replicative/infection kinetics correlated with tumor volumes after oHSV. Taking all three experiments together, there was a significant correlation after oHSV treatment or vehicle treatment between lower tumor volume and increased numbers in GP33+ CD8+ T cells (p < 0.001), gB498+ CD8+ T cells (p = 0.001) pan-CD4+ T cells (p = 0.019), but not pan-CD8+ T cells (p = 0.891) (Fig. 5). There were also strong negative and positive correlations between MRI, BLI and other cells of the immune compartment. For example, the GP33+ CD8+ T-cell population positively correlated with microglia (p < 0.001) and PMN-MDSCs (p = 0.002), while an increase in the CD4+ T-cell population correlated with oHSV variables (Fluc; p < 0.001, and gB498+ CD8+ T-cells; p < 0.001). There were also stronger correlations between the MDSC, macrophage, dendritic cell (DC), and microglia cell compartments and the GP33+ CD8+ T cell population than for the gB498+ CD8+ T cell population.

Correlations were also analyzed for each individual experiment (Figs. S8–S10). For experiment 1 (Fig. S8) there were no responders and thus as expected, there were no correlations between tumor response and antigen-specific T cells. As expected, oHSV activity, as measured by peak or area under the curve (AUC) Fluc, positively correlated with gB498+ CD8+ T cells (p = 0.007 and 0.01, respectively). Similarly, CD4+ T cells correlated with gB498+ CD8+ T cells (p = 0.019). In experiment 2 (Fig. S9) there was a significant correlation seen at 7 days after oHSV treatment between reduced tumor volumes and increased intratumoral numbers of GP33+ CD8+ T cells (p = 0.007), gB498+ CD8+ T cells (p = 0.007), CD4+ T cells (p = 0.032), as well as reduced numbers of M-MDSCs (p = 0.015). There was also an inverse correlation between M-MDSCs and both GP33+ and gB498+ CD8+ T cells (p = 0.009 and p = 0.007, respectively) CD8+ T cells. There were statistically significant negative correlations in oHSV variables (p = 0.041 for peak Fluc, and p = 0.001 for AUC Fluc) with tumor volumes. Similar results were also observed with MRI volumetric responses in experiment 3 (Fig. S10). There was a significant correlation between the reduction in tumor volume and increased populations of GP33+ CD8+ T cells (p = 0.038) or gB498+ CD8+ T cells (p = 0.014). These results suggest that several variables including the experimental setting of tumor cell numbers, viral doses and timing of tumor volume assessment impact the response to oHSV therapy. Consistently though, the most significant factors correlating with tumor reduction were the infiltration of both tumor and viral antigen-specific T cells and tumor oHSV gene expression levels.

## Discussion

The contribution of tumor antigen-specific infiltrating cytotoxic T cells to changes in MRI-measured volume of tumors after OV therapy has been assumed to be important but has not been formally proven, particularly in the context of oHSV and in the context of likely concomitant brisk antiviral cytotoxic T cell responses^4,5,8,24^. MRI volume-based outcome measures in cancer are routinely utilized as a non-invasive testing modality in cancer therapeutics to monitor the success of a therapy within patients over time but has not been extensively used in preclinical mouse cancer models. We hypothesized that oHSV administration in a mouse GBM model would lead to infiltration of both tumor antigen-specific and viral antigen-specific CD8+ T cells and that the former would correlate with a positive anticancer response as assayed by MRI reduction in tumor volume. We showed that: 1- mouse CT2A and GL261 GBM cells can be engineered to express high levels of human nectin-1 to allow for increased oHSV infectivity, and with a GP33 surrogate antigen to assay for T cell reactivity; 2- oHSV administration by stereotactic injection into mouse intracranial GBMs results in an increase in both tumor antigen specific and viral antigen specific CD8+ T cells in blood and tumors; 3- these T cells are functional, since they express IFN*γ* and do not over-express PD-1 or other markers of T cell “exhaustion”; 4- oHSV-mediated gene expression correlates with a reduction in tumor volume; and 5- infiltration of both tumor and viral antigen-specific CD8+ T cells correlates with a reduction in the MRI-measured volumes after oHSV treatment. Taken together, these data imply that positive anticancer efficacy of oHSV injection correlates with increases in both oHSV activity and with infiltration of functional tumor and viral-antigen specific CD8+ T cell responses.

Although the mouse GBM cell lines, CT2A and GL261, were engineered to express human nectin-1, they were not rejected when implanted into mouse brains. This may be because both mouse and human nectin-1 share 95% homology^25^ and thus the human gene product did not engender an immune response in the mouse. We did not try to express murine nectin-1 to see if it was as effective in allowing for oHSV entry and infection. We were also concerned that expression of the GP33 antigen would lead to tumor rejection, but this LCMV gene product was also fully tolerated in naïve mice, consistent with our previously published findings^16^. However, infection with oHSV did lead to expansion of GP33-specific CD8+ to a magnitude similar to that observed against the oHSV antigen gB498. This expansion occurred in both peripheral blood and intratumorally. This suggests that oHSV did infect the tumor mass and oHSV infection does indeed lead to activation of T cells against antigens other than just viral ones. The underlying mechanism may relate to enhanced presentation of tumor antigens due to the stress response and/or immunogenic cell death incurred by the infected cell^26–31^. In addition, priming efficacy of gB498+ T-cell may reflect the health of host immunity while failure of de novo T-cell activation could be associated with deficient expansion of pre-existing T-cells in response to the antigen exposure. However, it remains to be determined if this response can be maintained with endogenous tumor neoantigens, which may be presented or expressed in a weaker manner. The results shown in Fig. 3 suggest a general increase in a cytotoxic CD8+ T cell population that has low levels of PD-1 expression and that some of these cells may be reacting to tumor antigens other than GP33. Experiments are in progress to further define these issues.

It was also encouraging to observe that oHSV treatment led to infiltration of activated and functional cytotoxic CD8+ T cells, based on IFN*γ* expression and reduced PD-1 levels. The increase in CD8+ T cells was notable since these also failed to show an increase in PD-1, Tim-3, or Lag-3 when tumors were treated with oHSV compared to control. There was also an increased infiltration of CD4+ T cells that correlated with tumor reduction that is consistent with a supportive or “helper” role of CD4+ T cell to CD8+ T cells against both tumor and viral antigens. As shown by Saha *et al*.^24^, addition of immune checkpoint blockade may enhance oHSV therapy, although we did not see increased immune checkpoint signaling after oHSV treatment in our studies. This may relate to differences in tumor models or in the oHSVs that were utilized.

MRI-based volumetrics are routinely used clinically and in clinical trials to measure responses to therapy, but not as much in preclinical therapeutics studies in mice. It is somewhat interesting that tumors of the same volume in inbred mice do not all respond to the same dose of oHSV which suggests that there is tumor heterogeneity or that heterogeneous immune responses occur in different mice with the equivalent tumor volumes. We employed T2 sequences during MRI acquisition since they are technically less challenging than T1 with gadolinium, where mice tail veins have to be injected with the contrast agent. In the latter, uneven contrast acquisition leads to more variability in measurements and more mice have to be used since animal loss occurs from prolonged anesthesia.

The experiments shown in Fig. 2 illustrate that there exists a variability in immune response. This variability correlated the most with infiltration of CD8+ T cells that were specific for the GP33 and the gB498 antigens and with oHSV activity. Other parameters were not significant or were less significant. These results thus confirm that efficacy of oHSV therapy depends both on oHSV-mediated gene expression (infection and replication) and on infiltration of tumors by tumor antigen-specific as well as viral antigen-specific CD8+ T cells. This information is important since it affects future oHSV design. Our results suggest that an oHSV that promotes immunogenicity against tumor antigens and that can also replicate and lyse tumor cells effectively should yield the best results in tumor volume reduction and ultimately animal survival. It is clear though that OV antigens are also potent stimulators of CD8+ T cell expansion against viral antigens. In our experiments, these T cells also correlated with antitumor efficacy. However, since both GP33 and gB498 are immunodominant epitopes, one concern may be inefficient presentation of less immunodominant endogenous tumor antigens^17^. Additional experiments to address this question are in progress.

In summary, these findings confirm the need for both oHSV infection/replication and tumor- and viral antigen-specific CD8+ T cell tumor infiltration to reduce tumor volumes after injection.

## Methods

### Study approval

Tumor samples were obtained using a protocol approved by the Dana-Farber Cancer Institute IRB and performed in accordance with relevant guidelines and regulations. Written, informed consent was received from all participants prior to inclusion in the study. Animal studies were approved by Institutional Animal Care and Use Committee (IACUC) of the Brigham and Women’s Hospital Center for Comparative Medicine (BWH CCM) and performed in accordance with the guidelines and regulations of the BWH.

### Cell lines and culture

Murine and human glioma cell lines CT2A, CTgp33 GL261N4, U251 and U87*Δ*EGFR have been previously described^19,20^, and patient-derived primary GBM cells (BT145 and BT333) were obtained from DFCI’s CPDM (Center for Patient Derived Models)^32^. African green monkey Vero kidney cells were originally obtained from ATCC. CT2Agp33nectin1 were generated by transducing CTgp33 with Human Nectin1-expressing lentivirus vector as previously described^19^ and clonally selected from puromycin resistance (10 μg/ml) by fluorescence-activated cell sorting (FACS) with Nectin1 antibody (PE anti-human CD111 antibody, clone R1.302, Biolegend). Isolated clonal cells were further transduced with Rluc-expressing lentivirus and infected cells were selected using gentamycin and the clonal cell population confirmed using an in vitro cell lysate luciferase assay. Generation of these lentiviruses have been previously described^19^. Once the cell lines were established, expanded cells were cryopreserved to minimize passages before being used *in vivo*. All cell lines except the primary GBM cells were cultured as monolayers on adhesive culture dishes containing Dulbecco’s Modified Eagle Medium (DMEM; Thermo Fisher Scientific, Waltham, MA) supplemented with 10% fetal bovine serum (FBS, Sigma-Aldrich St. Louis, MO) and 100 U/ml penicillin-streptomycin (Thermo Fisher Scientific), 10 mM HEPES (4-(2-hydroxyethyl)-1-piperazineethanesulfonic acid) buffer (Thermo Fisher Scientific) at 37 °C in a humidified incubator maintained at 5% CO2. Primary glioma cells were maintained as neurospheres under stem cell conditions using Neurobasal media (Thermo Fisher Scientific) supplemented with GlutaMAX (Thermo Fisher Scientific), B27 (Thermo Fisher Scientific), 20 ng/ml epidermal growth factor (EGF) and fibroblast growth factor (FGF)-2 (PrepoTech Rocky Hill, NJ). Spheres were dissociated using StemPro Accutase Cell Dissociation Reagent (Thermo Fisher Scientific).

### Viral replication assay

For *in vitro* viral replication assay, 100,000 cells were cultured in 12-well plates containing corresponding growth media described earlier for 24 hours prior to the viral infection at an MOI of 0.1. BT145 and BT333 cells were cultured in plates coated with Poly D-lysine/Laminin solution (Sigma-Aldrich) to enhance cell adherence. Cells and medium were harvested at 24, 48, and 72 hours, followed by 3 cycles of freeze-thaw in dry ice-chilled ethanol, centrifuged at 300 g for 5 min. These supernatants were titered for viral yield on Vero cells as previously described^33^.

### *In vivo* animal studies

Six- to eight-week-old C57Bl/6 mice were purchased from Envigo (South Easton, MA). To establish the tumor in the mouse brain, dissociated CT2A, CT2Anectin1 or CT2Agp33nectin1 (200,000 cells in 5 μL of HBSS) were injected intracerebrally at stereotactic coordinates (ventral 3.5-mm, rostral 0.5-mm and right lateral 2.0-mm from the bregma using a stereotaxic apparatus (David Kopf Instruments). Cell numbers were varied in each experiment and are indicated in each specific figure legend. Intra-tumoral injection of 1 × 10^6^ pfu oHSV was performed on day 7 or 8. Mice were sacrificed on days 14, 15 or day 20 and brain tissues were harvested after perfusion with chilled PBS buffer. Brain tissues were dissociated with enzyme cocktail in RPMI1640 (Thermo Fisher Scientific) with 5% FBS at the following doses: 30 U/ml of DNase I type IV, 0.1 μg/ml of Hyaluronidase type V, and 1 μg/ml of Collagenase type IV (all from Sigma). Cells were then washed twice in RPMI with 2% FBS. Lymphocytes were enriched in the 67–44% Percoll gradient solution (GE Healthcare Chicago, IL) at 500 g for 40 minutes with zero deceleration at room temperature. Peripheral blood mononuclear cells (PBMCs) were enriched using Ficoll-Plaque Plus (GE Healthcare) at 1,900 rpm for 20 minutes. Single-cell suspensions from spleens were prepared by passing cells through 70-μm cell strainers and red blood cells were lysed using Ammonium-Chloride-Potassium (ACK) lysis buffer (Thermo).

### *In vivo* bioluminescence imaging

On day 6 or 7 following tumor implantation, fur was shaved off the head of the mice. Mice were anesthetized using isoflorane before and during bioluminescence imaging or MRI. For firefly luciferase/Fluc imaging, D-luciferin (Promega; dissolved in sterile D-PBS) at a dose of 3 mg per 20 g body weight was intraperitoneally injected in mice. For Renilla luciferase/Rluc imaging, 50 μg per 20 g body weight of Coelenterazine (Nanolight technology Pinetop, AZ; dissolved in sterile water) was injected intravenously via the tail vein. Light-emitted imaging was acquired with an IVIS Lumina LT with Living Image software (Perkin-Elmer, Waltham, MA) every 60 seconds, and images with peak signals were selected for analysis. Note that Rluc images were acquired at least 8 hours before Fluc images to avoid overlapping emission profiles during Fluc and Rluc imaging on the same day. Area under the curve (AUC) for correlation analysis was calculated with a baseline at 40,000 Photon/sec (p/s) using the AUC function in Prism (GraphPad Software; ver.7 or later).

### *In vivo* tumor volume measurement using magnetic resonance imaging (MRI)

MR images were acquired on indicated days after tumor implantation using the Bruker Biospec 3T Small Animal MRI equipment (BWH Research Imaging Core). To estimate tumor volume, we used the open-source software, Horos DICOM medical image viewer (Horos Project; ver. 3) to stack sliced T2-weighted images of each brain. Using a pencil tool, we highlighted tumor margins in each slice, excluding the obvious areas of brain edema, and the ROI volume tool was used to compute the tumor volume. However, we acknowledge that it is difficult to completely exclude brain edema in T2 sequences

### Antibodies and flow cytometry

For surface and intracellular staining, fluorophore-conjugated monoclonal antibodies (Abs) specific for CD45(30-F11), TCR*β*(H57-597), CD3 (17A2), CD4 (RM4-5), CD44 (IM7), CD279/PD-1 (RMP1-30), H-2D[b] (KH95), I-A[b] (AF6-120.1), TNF-*α* [MP6-XT22], IFN-*γ* (XMG1.2), IL2 (JES6-5H4), CD366/Tim-3 (B8.2C12), CD103 (2E7), Ly-6G (1A8), Ly-6C (HK1.4), CD11b (M1/70), CD115 (AFS98), F4/80 (BM8) were obtained from Biolegend (San Diego, CA), CD274/PD-L1 (MIH5), CD8a (53–6.7), TIGIT (1G9), CD223/LAG3 (C9B7W), CD11c (HL3) were from BD Biosciences (San Jose, CA). FoxP3 (MF-14), Live/Dead Near-IR Dead cell stain kit, and APC-streptavidin were from Thermo Fisher Scientific for GP33 and HSV gB498 tetramer staining, the biotinylated class I monomer, obtained from the National Institutes of Health Tetramer Core Facility (Emory University, GA), was conjugated with APC-streptavidin (GP33) or BV421-streptavidin (gB498) to form the tetramer. For intracellular IFN*γ* staining, 5 × 10^5^ Percoll-isolated tumor infiltrating lymphocytes were stimulated at 37 °C for 5 h with 1 × 10^5^ tumor cells or 1 μg/ml GP33-41 peptides (GenScript, Piscataway, NJ) in the presence of GolgiStop and GolgiPlug (BD Bioscience). Intranuclear FoxP3 staining was performed with the eBioscience Foxp3 Transcription Factor Staining Buffer Set (Thermo Fischer Scientific), following the manufacturer’s instructions. Following staining, cells were fixed using 2% formaldehyde and were run on an LSR II (BD Biosciences) at the CCVR Flow Cytometry Core in Beth Israel Deaconess Medical Center (BIDMC; Boston, MA), and analyses were performed with FlowJo (TreeStar; ver. 10). Gating strategies are shown in Fig. S5.

### Statistics

Statistical analysis was performed with Prism (GraphPad Software; version 7 or later) and R (version 3.5.0 or later). All p-values are reported, p-values < 0.05 were considered significant. For continuous variables, such as tumor volume, ANOVA models with repeated measures, or linear mixed effects models, or a paired t-test unless otherwise indicated were used and p-values were corrected for multiple comparisons. Kaplan–Meier analysis was performed using log-rank Mantel-Cox test with correction for multiple comparisons (Holm’s post-test). For the linear regression and correlation analysis, all data were converted into log10 scale to reduce skewness and variance. Color gradient was used to indicate the strength of correlation and p-value levels in Fig. 5 and Figs. S8–S10 were generated using a color scale function in Microsoft Excel (ver. Office365 E3). Final graphs and tables were formatted using Adobe Illustrator (ver. CS6).

## Supplementary information

Supplementary Information.
