# Supplementary material for: Glioblastoma infiltration of both tumor- and virus-antigen specific cytotoxic T cells correlates with experimental virotherapy responses
Source: Sci Rep. 2020 Mar 20;10:5095. doi: 10.1038/s41598-020-61736-2 (PMC7083912; doi:10.1038/s41598-020-61736-2)
Supplement: Supplementary file 1 — Supplementary Information. [file 41598_2020_61736_MOESM1_ESM.pdf]

Supplementary Data

Glioblastoma infiltration of both tumor- and virus-antigen specific cytotoxic T cells correlates with experimental virotherapy responses

Quazim A. Alayo[1], Hirotaka Ito[1], Carmela Passaro[1], Mykola Zdioruk[1], Ahmad Bakur Mahmoud[1,2], Korneel Grauwet[1,3], Xiaoli Zhang[4], Sean E. Lawler[1], David A. Reardon[5], William F. Goins[6], Soledad Fernandez[4], E. Antonio Chiocca[1,7], Hiroshi Nakashima[1,7]

[1] Harvey W. Cushing Neuro-oncology Laboratories (HCNL), Department of Neurosurgery, Harvard Medical School and Brigham and Women's Hospital, Boston, MA, 02115, USA.

[2] Current address: College of Applied Medical Sciences, Taibah University, Madinah, 42353, Saudi Arabia.

[3] Current address: Massachusetts General Hospital, Cancer Center and Department of Medicine, Harvard Medical School, Boston, Massachusetts USA

[4] Center for Biostatistics, Department of Biomedical Informatics, The Ohio State University, Columbus, OH, 43210, USA

[5] Center for Neuro-Oncology, Dana-Farber Cancer Institute Boston, MA, 02115, USA.

[6] Department of Microbiology and Molecular Genetics, University of Pittsburgh School of Medicine, Pittsburgh, PA 15219, USA.

[7] Correspondence to: EA Chiocca eachiocca@bwh.harvard.edu and H. Nakashima hnakashima@bwh.harvard.edu

Figure S1– Representative histogram showing frequency of human nectin-1 expression (Y-axis) on CT2A (white) and CT2Anectin1 (grey) cells by florescence activated cell sorting analysis (FACS) using anti-human nectin-1/CD111 antibody (**a**). Representative fluorescence microscopy images (**b**) of CT2A and CT2Anectin1 at 24hrs and 48hrs infected with GFP-expressing oHSV at a MOI of 0.1. CT2A and CT2Anectin1 were infected at the indicated MOIs and cell survival was analyzed 6 days after infection using the CellTiter-Glo® 2.0 Cell Viability Assay (**c**). The viral Lethal Dose 50 (LD_{50}) shown in the table was calculated using a sigmoidal dose response curve. C57Bl/6 mice were intracranially injected with CT2A or CT2Anectin1 cells (2×10^5 cells) and survival was assessed (**d**). Kaplan-Meier survival curves after tumor cell implantation were analyzed with Gehan-Breslow-Wilcoxon test, $p = 0.01$.

Figure S1– Representative individual MRIs and BLIs of responders vs. non-responders to oHSV therapy. $2x10^5$ CT2Agp33nectin1 GBM cells were implanted in the brains of C57Bl/6 mice. Seven days later, $2x10^6$ pfu of oHSV expressing Fluc were injected stereotactically into the tumor. (**Panels a-m**) Pre- and post- oHSV treatment MRI images are shown for 3 responders (decreased or stable tumor volume; **panel a-f**) and 2 non-responders (increased tumor volume; **panel j-m**). White arrows point to right frontal brain tumor in MRIs. MRI images are T2 weighted. (**Panels g-o)** Representatives plots of BLI and MRI-measured tumor volumes and BLI-measured oHSV replication for the same 3 responder **(panels g-i)** and 2 non-responder **(panels n,o)** mice whose corresponding MRIs are in **panels a-m** . BLI are for Fluc-based oHSV replicative kinetics (red line and circles) and Rluc-based tumor viability (blue line and squares). MRI-measured tumor

volumes are shown with black lines and triangles. Gray dotted lines indicate the level of background photon noise using BLI.

Figure S3– Single mouse MRI and BLI data for the 3 *in vivo* experiments. **a)** Schematic describes the timing of oHSV treatment, MRI imaging and tissue harvest for FACS analysis after tumor implant (day 0). **Panels b, e, h)** tumor volumes (mm^3) pre- and post-treatment for each mouse in the 3 experiments were measured by MRI and plotted with bars and lines. **Panels c, f, i)** Fluc and **panels d, g, j)** Rluc bioluminescent imaging for each mouse in the 3 experiment were measured and plotted on the on y-axis in a logarithm scale vs. indicated days on the x-axis. Statistical analyses used linear mixed effects models into account the correlation among the observations from the same mouse as follows: The results showed that the increase tumor volume measured for the oHSV treated group was significantly lower than that of the vehicle treated control group in experiment 2 (p-value=0.004) **(panel g)**, but the difference in change in the tumor volume between the two groups was not significant in experiments 1 and 3 (p=0.65 and 0.068, **panels d** and **j** respectively). The trend in tumor growth over time was estimated and compared between oHSV treated and control groups from this model. The results showed that tumor growth over time was significantly slower in experiment 2 comparing oHSV to vehicle control (p-value=0.0004, **panel g**), but the rate of tumor growth between the groups were not significant in experiments 1 and 3 (p=0.57 and 0.31, **panels d** and **j,** respectively). The trend in the change in viral yield over time was estimated from this model. The results showed that for all three experiments, the viral yield was significantly decreased over time (p-values=0.0005, <.0001, and 0.0021 for experiments 1, 2, and 3, **panels c, f, i,** respectively,).

Figure S4 – Comparison of pre-oHSV treatment tumor volumes measured by MRI between experiment 1 and experiments 2 and 3. Tumor volume was assessed by MRI on day 6 or 7 in all three experiments, and this graph showed that there was no difference between pre-treatment tumor volumes in experiment 1 and experiments 2 and 3 (p=0.1714).

Figure S5- Flow cytometry gating strategies for T cells and myeloid cells in tumor-infiltrating lymphocytes (TILs). Antibodies and flow cytometry methods are described in the Materials and Methods section of main text. Live lymphocytes were identified using Live-dead -ve (negative) CD45 +ve (positive) cells. T cells were identified using Tcrβ +ve cells. Other subsets of T cells were identified as shown in (**a**). Different subpopulations of myeloid cells were identified as shown in (**b**).

Figure S6– Percentage (**a**) and Number (**b**) of total CD8+ T cells recruited to the mouse brains on day 7 after oHSV (triangles) or PBS vehicle injection (squares) in orthotopic CTgp33N5 GBMs.

Figure S7– Summary of mean fluorescence intensity (MFI) of immune-inhibitory molecules (PD-1, LAG-3, Tim-3, and TIGIT) expression of the surface of CD8+ T cells recruited to the mouse brains on day 7 after oHSV or PBS injection into orthotopic CTgp33N5 GBMs.

Figure S8, Figure S9, Figure S10– Pearson's correlation matrix for each experiment used in **Figure 6** comparing MRI, BLI and FACS data. Data were analyzed separately for each experiment. n indicates sample number.

Figure S1

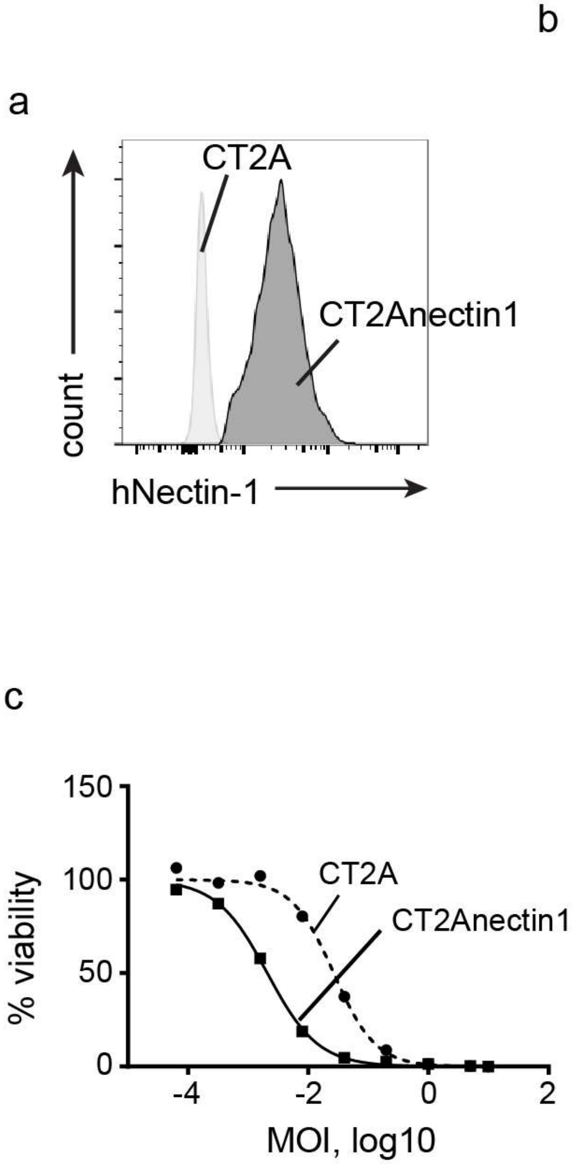

Figure S2

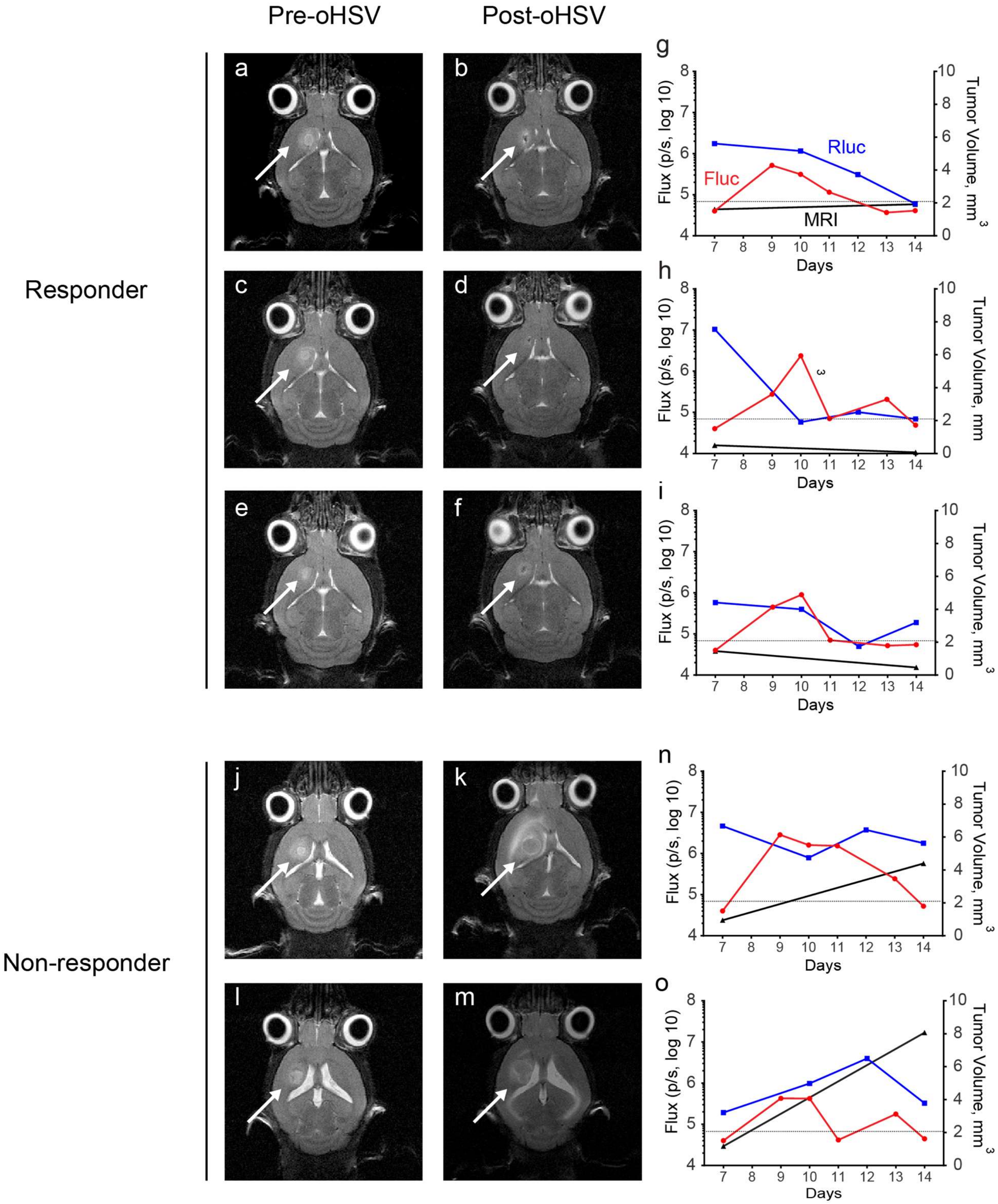

Pre-oHSV
Post-oHSV
Responder
Non-responder
a
b
c
d
e
f
g
h
i
j
k
l
m
n
o
Flux (p/s, log 10)
Tumor Volume, mm 3
Days
Rluc
Fluc
MRI

Figure S3

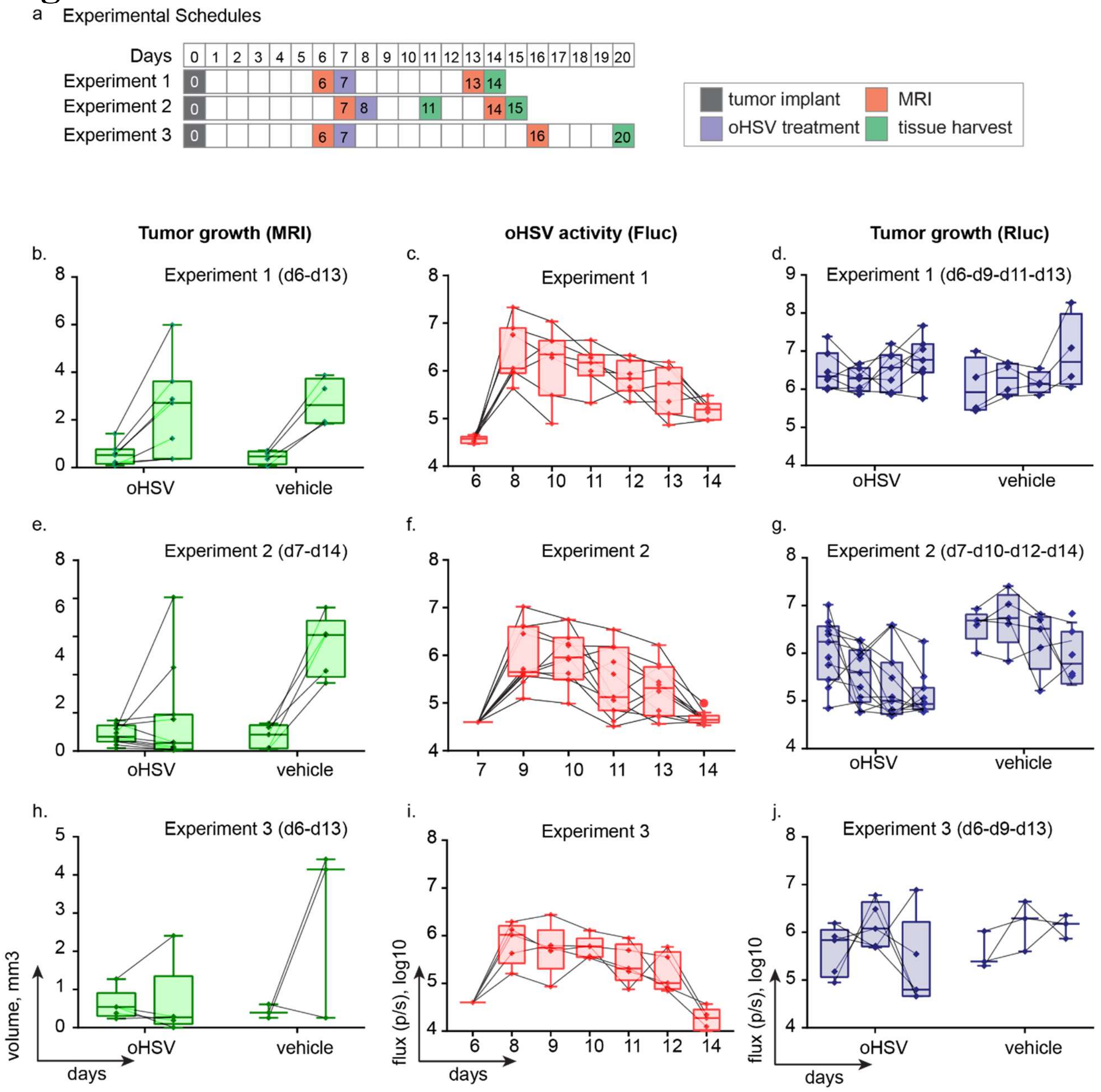
a
Experimental Schedules
Days
Experiment 1
Experiment 2
Experiment 3
tumor implant
MRI
oHSV treatment
tissue harvest
Tumor growth (MRI)
oHSV activity (Fluc)
Tumor growth (Rluc)
b.
Experiment 1 (d6-d13)
c.
Experiment 1
d.
Experiment 1 (d6-d9-d11-d13)
e.
Experiment 2 (d7-d14)
f.
Experiment 2
g.
Experiment 2 (d7-d10-d12-d14)
h.
Experiment 3 (d6-d13)
i.
Experiment 3
j.
Experiment 3 (d6-d9-d13)
oHSV
vehicle
volume, mm3
flux (p/s), log10
days

Figure S4

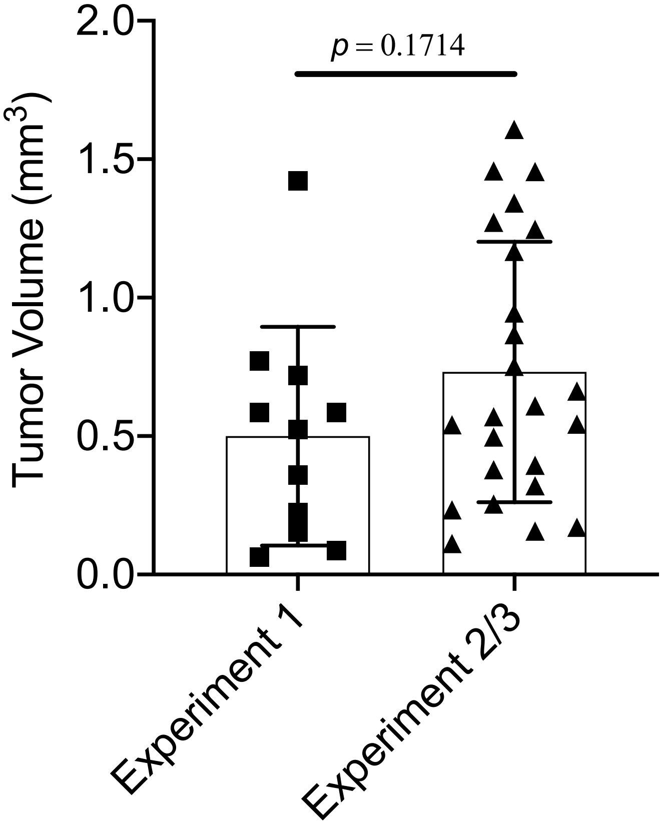

p = 0.1714
Tumor Volume (mm3)
2.0
1.5
1.0
0.5
0.0
Experiment 1
Experiment 2/3

Figure S5

a

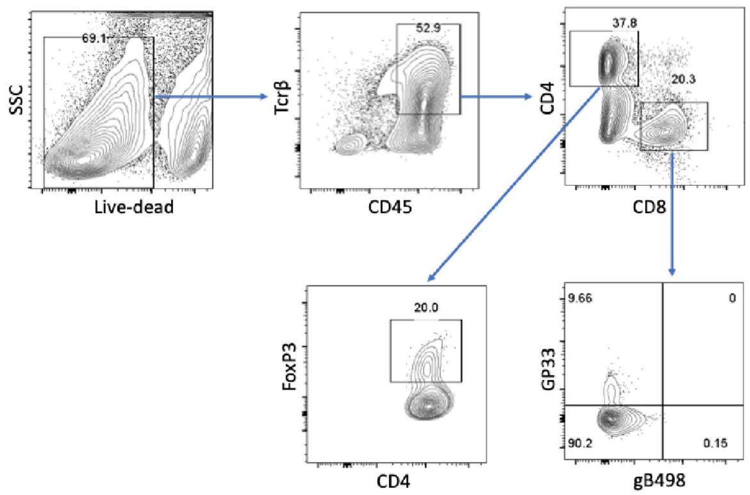

69.1
SSC
Live-dead
52.9
Tcrβ
CD45
37.8
20.3
CD4
CD8
20.0
FoxP3
CD4
9.66
0
90.2
0.15
GP33
gB498

b

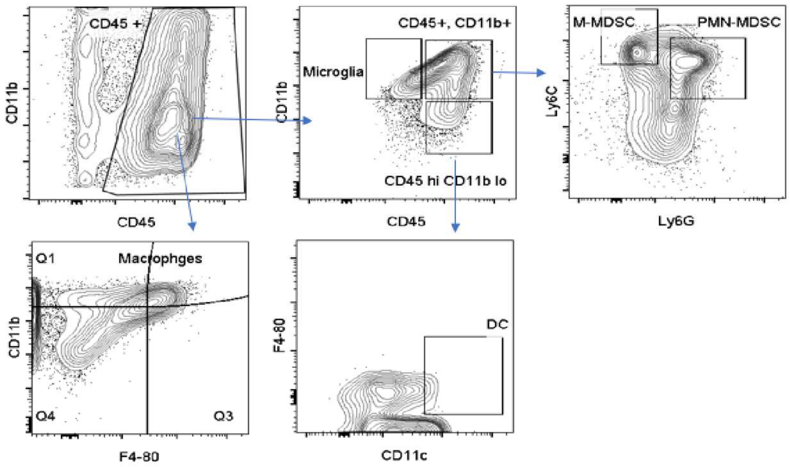

CD45 +
CD11b
CD45
CD45+, CD11b+
Microglia
CD45 hi CD11b lo
CD11b
CD45
M-MDSC
PMN-MDSC
Ly6C
Ly6G
Q1
Macrophges
Q4
Q3
CD11b
F4-80
DC
F4-80
CD11c

Figure S6

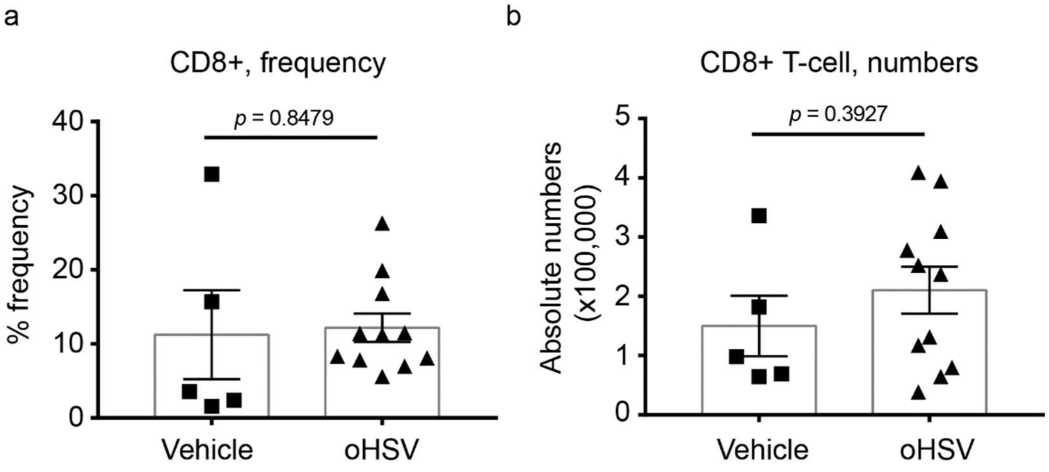

a
CD8+, frequency
p = 0.8479
% frequency
40
30
20
10
0
Vehicle
oHSV
b
CD8+ T-cell, numbers
p = 0.3927
Absolute numbers (x100,000)
5
4
3
2
1
0
Vehicle
oHSV

Figure S7

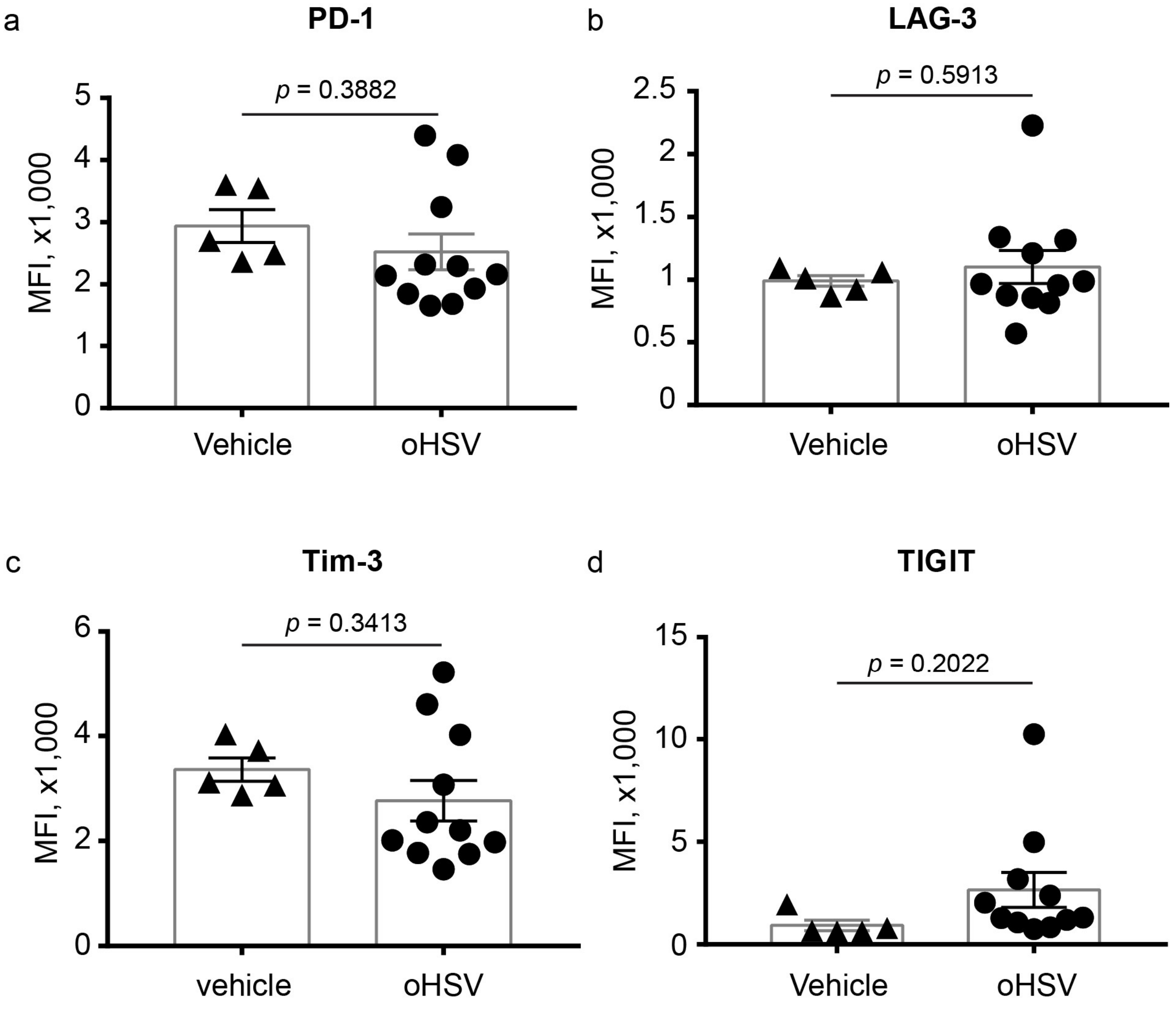
a
PD-1
p = 0.3882
MFI, x1,000
0
1
2
3
4
5
Vehicle
oHSV
b
LAG-3
p = 0.5913
MFI, x1,000
0
0.5
1
1.5
2
2.5
Vehicle
oHSV
c
Tim-3
p = 0.3413
MFI, x1,000
0
2
4
6
vehicle
oHSV
d
TIGIT
p = 0.2022
MFI, x1,000
0
5
10
15
Vehicle
oHSV

Figure S8

Experiment 1 (n=11)

	MRI		BLI				FACS									
	MRI: pre	MRI: post	Peak Rluc	AUC Rluc	Peak Fluc	AUC Fluc	CD8+	GP33+CD8+	gB498+CD8+	CD4+	FoxP3+CD4+	Microglia	PMN-MDSC	M-MDSC	DC	Macrophage
MRI: pre	—	0.662	0.334	0.366	0.014	0.039	-0.363	-0.441	0.176	-0.003	-0.146	-0.253	-0.358	0.452	0.672	0.08
MRI: post	0.026	—	0.579	0.214	-0.356	-0.358	-0.339	-0.517	0.009	-0.283	0.316	-0.071	-0.091	-0.042	0.376	0.251
Peak Rluc	0.315	0.062	—	0.675	0.254	0.266	-0.159	-0.488	0.311	0.187	-0.261	-0.365	-0.519	0.292	-0.052	0.196
AUC Rluc	0.268	0.528	0.023	—	0.292	0.372	-0.171	-0.243	0.226	0.199	-0.312	-0.249	-0.452	0.574	-0.068	0.316
Peak Fluc	0.967	0.282	0.451	0.384	—	0.992	-0.191	-0.364	0.76	0.913	-0.904	-0.809	-0.638	0.367	0.096	-0.621
AUC Fluc	0.91	0.279	0.428	0.259	< .001	—	-0.205	-0.357	0.735	0.912	-0.911	-0.791	-0.679	0.423	0.103	-0.575
CD8+	0.272	0.308	0.64	0.615	0.575	0.546	—	0.748	-0.674	-0.129	0.033	0.285	0.134	-0.065	-0.446	0.055
GP33+CD8+	0.175	0.103	0.128	0.471	0.271	0.281	0.008	—	-0.744	-0.364	0.29	0.59	0.453	-0.273	-0.539	0.179
gB498+CD8+	0.605	0.979	0.351	0.505	0.007	0.01	0.023	0.009	—	0.689	-0.621	-0.77	-0.471	0.219	0.387	-0.474
CD4+	0.993	0.399	0.583	0.557	< .001	< .001	0.704	0.271	0.019	—	-0.903	-0.906	-0.677	0.202	0.267	-0.789
FoxP3+CD4+	0.669	0.344	0.438	0.35	< .001	< .001	0.924	0.387	0.041	< .001	—	0.799	0.777	-0.474	-0.284	0.594
Microglia	0.454	0.836	0.269	0.461	0.003	0.004	0.396	0.056	0.006	< .001	0.003	—	0.596	-0.124	-0.408	0.773
PMN-MDSC	0.28	0.79	0.102	0.163	0.035	0.022	0.693	0.161	0.144	0.022	0.005	0.053	—	-0.657	-0.443	0.214
M-MDSC	0.163	0.904	0.384	0.065	0.266	0.194	0.85	0.416	0.518	0.552	0.141	0.716	0.028	—	0.273	0.307
DC	0.024	0.254	0.879	0.843	0.779	0.763	0.169	0.087	0.239	0.428	0.397	0.213	0.173	0.417	—	-0.284
Macrophage	0.815	0.457	0.563	0.343	0.042	0.064	0.871	0.598	0.141	0.004	0.054	0.005	0.527	0.359	0.398	—

p-value < 0.001 – 0.05

correlation coefficient / p-value

Pearson's Correlation -1.0 0 1.0

Figure S9

Experiment 2 (n=16)

	MRI		BLI				FACS									
	MRI: pre	MRI: post	Peak Rluc	AUC Rluc	Peak Fluc	AUC Fluc	CD8+	GP33+CD8+	gB498+CD8+	CD4+	FoxP3+CD4+	Microglia	PMN-MDSC	M-MDSC	DC	Macrophage
MRI: pre	—	0.291	0.152	0.127	-0.697	-0.149	0.397	-0.058	0.015	0.01	0.328	0.067	-0.089	0.06	-0.108	0.187
MRI: post	0.274	—	0.679	0.725	-0.622	-0.734	0.173	-0.648	-0.649	-0.537	0.083	-0.453	-0.259	0.594	-0.135	-0.202
Peak Rluc	0.574	0.004	—	0.899	-0.57	-0.707	0.317	-0.44	-0.608	-0.554	-0.038	-0.454	-0.23	0.54	-0.178	-0.275
AUC Rluc	0.639	0.001	< .001	—	-0.321	-0.599	0.335	-0.463	-0.707	-0.597	0.052	-0.376	-0.309	0.555	-0.066	-0.187
Peak Fluc	0.017	0.041	0.067	0.336	—	0.979	-0.544	0.262	0.363	0.557	0.09	0.465	0.067	-0.518	0.369	0.362
AUC Fluc	0.583	0.001	0.002	0.014	< .001	—	-0.322	0.464	0.702	0.76	0.299	0.405	0.024	-0.546	0.004	0.443
CD8+	0.128	0.522	0.231	0.205	0.084	0.224	—	0.033	-0.453	-0.439	0.049	-0.569	-0.314	0.471	-0.373	0.021
GP33+CD8+	0.832	0.007	0.088	0.071	0.436	0.07	0.903	—	0.6	0.653	-0.079	0.512	0.365	-0.63	0.35	0.308
gB498+CD8+	0.956	0.007	0.013	0.002	0.273	0.002	0.078	0.014	—	0.845	0.234	0.634	0.261	-0.641	0.038	0.147
CD4+	0.97	0.032	0.026	0.015	0.075	< .001	0.089	0.006	< .001	—	0.214	0.547	0.251	-0.799	0.209	0.471
FoxP3+CD4+	0.214	0.761	0.888	0.849	0.791	0.261	0.858	0.771	0.383	0.427	—	0.085	-0.689	-0.067	-0.252	0.375
Microglia	0.807	0.078	0.077	0.151	0.15	0.12	0.021	0.043	0.008	0.028	0.755	—	0.404	-0.742	0.663	0.133
PMN-MDSC	0.743	0.333	0.39	0.244	0.844	0.93	0.237	0.164	0.329	0.349	0.003	0.12	—	-0.334	0.357	-0.29
M-MDSC	0.825	0.015	0.031	0.026	0.103	0.028	0.066	0.009	0.007	< .001	0.805	< .001	0.206	—	-0.585	-0.425
DC	0.69	0.619	0.509	0.809	0.264	0.987	0.155	0.184	0.888	0.438	0.346	0.005	0.174	0.017	—	0.295
Macrophage	0.487	0.453	0.303	0.488	0.275	0.086	0.939	0.247	0.588	0.065	0.152	0.624	0.276	0.101	0.267	—

p-value < 0.001 — 0.05

Upper triangle: correlation coefficient; lower triangle: p-value

Pearson's Correlation -1.0 — 0 — 1.0

Figure S10

Experiment 3 (n=8)

	MRI		BLI				FACS									
	MRI: pre	MRI: post	Peak Rluc	AUC Rluc	Peak Fluc	AUC Fluc	CD8+	GP33+CD8+	gB498+CD8+	CD4+	FoxP3+CD4+	Microglia	PMN-MDSC	M-MDSC	DC	Macrophage
MRI: pre	—	0.083	0.55	0.435	0.15	-0.08	-0.244	-0.151	-0.349	0.119	-0.182	-0.489	-0.662	0.722	0.544	-0.429
MRI: post	0.86	—	0.435	0.354	0.093	-0.181	0.071	-0.782	-0.857	-0.526	0.229	-0.414	-0.415	0.353	0.565	-0.468
Peak Rluc	0.158	0.329	—	0.368	0.022	-0.309	-0.352	-0.096	-0.431	0.1	0.077	-0.141	-0.328	0.496	0.397	-0.196
AUC Rluc	0.282	0.436	0.369	—	-0.129	-0.153	0.307	0.088	-0.191	0.207	-0.091	0.146	-0.006	-0.119	-0.068	0.122
Peak Fluc	0.81	0.907	0.972	0.836	—	0.94	-0.613	-0.575	-0.316	0.362	-0.168	0.365	0.323	0.223	0.45	-0.016
AUC Fluc	0.898	0.819	0.613	0.806	0.018	—	-0.371	-0.519	-0.122	0.252	-0.205	0.539	0.512	-0.083	0.227	0.111
CD8+	0.56	0.88	0.392	0.46	0.271	0.539	—	0.322	-0.05	-0.496	0.647	-0.085	-0.005	-0.353	-0.475	0.542
GP33+CD8+	0.722	0.038	0.821	0.835	0.311	0.37	0.436	—	0.783	0.467	0.061	0.529	0.439	-0.566	-0.791	0.761
gB498+CD8+	0.396	0.014	0.287	0.65	0.604	0.845	0.906	0.022	—	0.703	-0.428	0.663	0.639	-0.681	-0.713	0.474
CD4+	0.779	0.225	0.813	0.622	0.55	0.682	0.211	0.243	0.052	—	-0.78	0.661	0.549	-0.382	-0.243	0.124
FoxP3+CD4+	0.666	0.622	0.856	0.83	0.787	0.741	0.083	0.885	0.291	0.022	—	-0.282	-0.233	0.109	-0.127	0.44
Microglia	0.219	0.356	0.739	0.731	0.545	0.349	0.841	0.178	0.073	0.074	0.499	—	0.932	-0.835	-0.676	0.649
PMN-MDSC	0.074	0.354	0.428	0.988	0.596	0.378	0.991	0.276	0.088	0.159	0.579	< .001	—	-0.884	-0.595	0.624
M-MDSC	0.043	0.437	0.212	0.779	0.719	0.895	0.391	0.144	0.063	0.351	0.797	0.01	0.004	—	0.833	-0.698
DC	0.163	0.186	0.33	0.873	0.447	0.713	0.234	0.019	0.047	0.561	0.765	0.066	0.12	0.01	—	-0.804
Macrophage	0.289	0.29	0.642	0.773	0.98	0.859	0.165	0.028	0.235	0.77	0.276	0.082	0.098	0.054	0.016	—

p-value < 0.001 – 0.05

Upper triangle: correlation coefficient; lower triangle: p-value

Pearson's Correlation -1.0 0 1.0
